# Experimental investigation and modelling of a laboratory-scale latent heat storage with cylindrical PCM capsules

**DOI:** 10.1038/s41598-021-02705-1

**Published:** 2021-12-01

**Authors:** Petr Jančík, Michal Schmirler, Tomáš Hyhlík, Adam Bláha, Pavel Sláma, Jakub Devera, Jan Kouba

**Affiliations:** Department of Fluid Dynamics and Thermodynamics, Faculty of Mechanical Engineering, Czech Technical University in Prague, Technická 4, 160 00 Prague, Czech Republic

**Keywords:** Energy storage, Mechanical engineering, Energy science and technology, Engineering

## Abstract

Heat storage efficiency is required to maximize the potential of combined heat and power generation or renewable energy sources for heating. Using a phase change material (PCM) could be an attractive choice in several instances. Commercially available paraffin-based PCM was investigated using T-history method with sufficient agreement with the data from the manufacturer. The introduced LHTES with cylindrical capsules is simple and scalable in capacity, charging/discharging time, and temperature level. The overall stored energy density is 9% higher than the previously proposed design of similar design complexity. The discharging process of the designed latent heat thermal energy storage (LHTES) was evaluated for two different flow rates. The PCM inside the capsules and heat transfer fluid (HTF) temperature, as well as the HTF flow rate, were measured. The lumped parameter numerical model was developed and validated successfully. The advantage of the proposed model is its computational simplicity, and thus the possibility to use it in simulations of a whole heat distribution network. The so-called state of charge (SoC), which plays a crucial role in successful heat storage management, is a part of the evaluation of both experimental and computational data.

## Introduction

Short term thermal energy accumulation can be an effective way for optimizing combined heat power (CHP) units and district heating (DH) networks. For a CHP unit, heat storage allows decoupling heat and electricity distribution and thus increases earnings^1,2^. For a DH system, some benefits of energy storing are smaller distribution pipes^3^, and more efficient utilization of heat sources due to peak shaving and valley filling^4^.

Nowadays, the most common medium for short-term heat storage is water. The main reasons are its availability, favourable thermal properties, and it usually serves as a heat transfer fluid (HTF), so heat exchangers are not necessary. However, a drawback is that a relatively high temperature difference has to be achieved for sufficient energy density^4^. A latent heat thermal energy storage (LHTES) tackles this disadvantage by using phase change materials (PCMs). PCMs are substances that change their phase (between liquid and solid usually) in a narrow temperature range and this change is associated with significant thermal energy release or absorption. It seems that LHTS can be cost effective in combination with micro-CHP units when compared to water sensible heat storage thanks to its size reduction^5^.

PCMs used in LHTESs are classified into several categories from the chemical point of view^6^. PCMs based on salt hydrates, paraffins, fatty acids, or other organic compounds are commonly used, but rarely as pure substances. Various additives improve their properties such as long-term stability or heat conductivity. Currently, several companies offer a variety of PCMs for many purposes. Rubitherm, Climator, or PCM Products, to name just a few of them. Salt hydrates have the advantages of high density, high energy density, and relatively high thermal conductivity. However, phase separation that decreases storage capacity can occur, and subcooling complicates their use as well^7^. Even though some additives were proposed to combat phase separation^6^, it remains a problem until today^7^. Salt hydrates are also corrosive on metals^6^. Parafins, on the other hand, have relatively low stored energy density and lower thermal conductivity. Advantages of paraffin are long-term stability and no subcooling^6^.

PCM thermodynamic properties must be known precisely for designing a LHTES. The phase change enthalpy and the phase change temperature are the most important properties. Differential scanning calorimetry (DSC) or differential thermal analysis (DTA) are standard methods for obtaining the results. However, these methods use very small material samples (order of 10μL) which causes problems with heterogenous materials and the sample size affects some characteristics of PCMs, such as subcooling^9^. More appropriate for investigating PCMs seems to be T-history method proposed by Yinping and Yi^10^, which was later improved by Marín et al.^11^ or Kravvaritis et al.^12^. The ability to measure multiple experimental samples of PCM at the same time is the method's key advantage. It also enables for a relatively large sample of PCM (required for salt hydrate PCM) to be examined in comparison to commercial methods and the construction of an experimental set-up is cost effective^9^.

HTF and PCM have to be separated, but heat has to be exchanged between them intensively at the same time. PCM can be encased in capsules of various sizes and shapes^13^. Nuitten et al. compared small spherical capsules with paraffin PCM and larger cylindrical capsules made of HDPE with salt hydrate PCM^14^. Xu et al. used commercially available slab and ellipsoid macrocapsules filled with paraffin PCM^15^. Xu et al. filled HDPE cylinders with salt hydrate^8^. Designs originating from heat exchangers were investigated as well. Medrano et al. filled by paraffin based PCM some commercially available heat exchangers and a shell-and-tube heat exchanger with copper fins or graphite matrix^16^. Zauner et al. designed a fin-tube storage with HDPE as the PCM^17^. Zauner et al. used shell-and-tube design for their sensible-latent heat storage^18^. Various heat transfer enhancement techniques have been proposed. Fins of various positions, shapes, and sizes were studied by many researchers, mostly in shell-and-tube configurations^19,20^. Using metal, carbon, or graphite foams can increase effective heat conduction of PCMs significantly^21,22^. However, these materials are costly and therefore usually not applicable in larger scales. A budget alternative for metallic foams might be brushes or chips^23^. There is no clear consensus on the ideal design of a LHTES, because many factors must be considered, and the requirements are often contradictive. Especially the need of shortening charging and discharging periods results in more complicated and thus costly solutions.

Some numerical models have lately been released, mainly as virtual replicas of physical devices. Barz et al. used FVM (finite volume method) in cylindrical coordinates to describe their shell and tube latent heat storage^24^. In ANSYS Fluent, Zauner et al. constructed an FVM model of their fin-tube heat storage^17^. In 2017, Zauner et al. developed a hybrid sensible-latent heat storage system that was modelled in Dymola as the Stefan problem with lumped capacity and variable specific heat^18^. COMSOL FEM software was used by Xu et al. to model cylindrically encapsulated latent heat storage^25^. Talati and Taghilou used the lattice Boltzmann method for PCM solidification in 2D domains and found that it is significantly more computationally efficient that the FVM^26^. Majority of the proposed models are 2D and some of them are 1D or 3D^27^. They are usually computationally expensive and not suitable for integration into larger models of whole distribution systems.

It is critical to know how much energy may be consumed or stored at any time when operating a heat storage system. It is a relatively straightforward process for sensible heat storage, given the dependence of enthalpy on temperature for the bulk of accumulation media, such as water, is well understood. State of charge (SoC) is commonly employed as a charge measurement for latent heat storage. SoC, on the other hand, does not have a singular definition^28,30^. Some publications solely include the PCM's latent heat^24^, while others consider its sensible heat and energy stored in additional storage components^28^. There is a variety of measurement approaches that can be used to obtain the data required for SoC evaluation. The most obvious method is to directly measure the temperature of the PCM or calculate enthalpy^28^. However, because the temperature field is not uniform, this usually requires many sensors. It is also feasible to take advantage of PCM’s volume changes during the phase transition. When the volume filled with the PCM is closed, pressure differences can be detected^28,31,32^. This method requires fewer sensors, but it fails to evaluate sensible heat because it is coupled with too minor volume changes. HTF temperature measurement is another option, which is reasonably straightforward but does not easily account for heat losses that impact storage capacity over time^28^.

This article presents the design of a latent heat storage on a laboratory scale that can be simply scaled to fit its desired purpose in temperature, size, and cycle period time. The design simplicity allowed the development of a simple and computationally inexpensive numerical model, which used a temperature-dependent specific heat capacity model for the PCM. Two processes of discharge with different heat transfer fluid flow rates were conducted and the data from these experiments were used for validation of the numerical model and for deeper understanding of the solidification process within the cylindrical capsules and discharging the storage. State of charge (SoC), an important parameter for monitoring the storage, was evaluated in two different ways from the experimental and numerical data as well.

In the end of this section, it is worth to emphasise several novel features, which starkly distinguish the LHTES considered in the presented study among existing prototypes. First, although very simple, the design considered here provides a very high value of the stored energy density, while guaranteeing very short spans of the discharging time necessary for high storage flexibility. All this is achieved together with a high level of scalability of design. Furthermore, as can be seen from the results of experimental investigation, the LHTES considered here performed very well in comparison with other storages. In addition, the novel numerical model, developed in this study, is computationally very effective and yet sufficiently precise.

## PCM specific enthalpy investigation

It is crucial to understand the thermophysical properties of the materials utilized when modelling a thermal storage with PCM. For commonly used media and materials (water, stainless steel), their qualities are well known, and their uncertainty plays a little role overall. However, PCM has the biggest impact on the outcomes of tests and simulations, even though the data's veracity is debatable.

### T-history method

The authors did not want to depend just on the information provided. As a result, an apparatus for determining partial enthalpy using the T-history approach was built, and RT35HC^29^ was measured in it. T-history is an experimental method created by Yinping and Yi^10^, and the initial configuration allowed for the measurement of melting point, heat of fusion, degree of subcooling, thermal conductivity, and specific heat capacity. The data processing method introduced by Marín et al.^11^ was used in this work.

**Figure 1:**
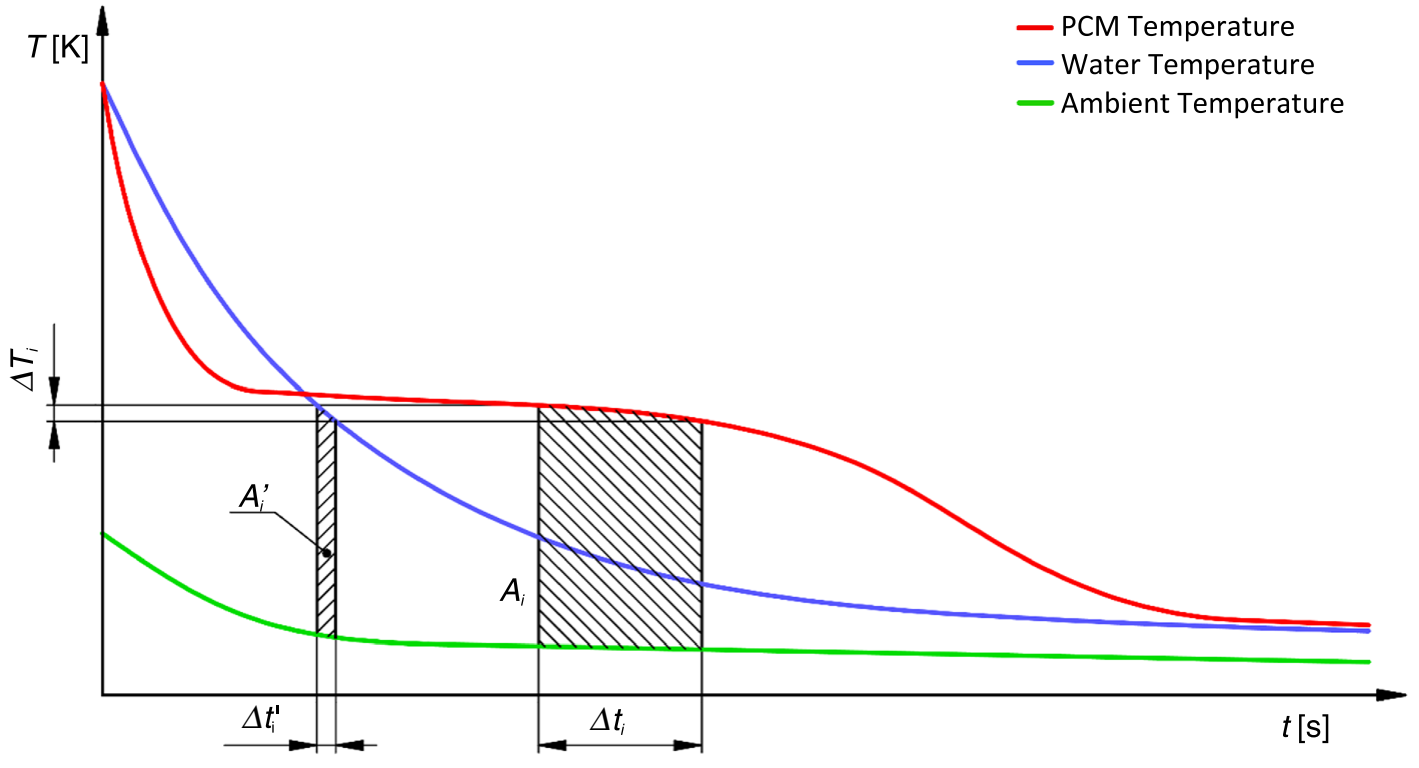
T-history method temperature–time curves (Marín method), demonstration of ${A}_{i}$ and ${A}_{i}^{^{\prime}}$ coefficients.

The T-history method uses the temperature evolution of two (or more) samples in test tubes, one with known properties (typically water) and one (or more) with unknown attributes. All samples are heated to the same temperature before being placed in a chamber with a variable temperature. The temperature evolution is used to analyse the unknown attributes. Figure 1 depicts a typical temperature evolution.

The algorithm described by Marín et al.^11^ was used to calculate the partial enthalpy, which may be compared to data from Rubitherm. It yields

1 $${h}_{part}\left(T\right)=\frac{{m}_{w}\cdot {c}_{pw}\left(T\right)+{m}_{t}\cdot {c}_{pt}\left(T\right)}{{m}_{PCM}}\cdot \frac{A}{{A}^{^{\prime}}}\cdot \Delta T-\frac{{m}_{t}}{{m}_{p}}\cdot {c}_{pt}\left(T\right)\cdot \Delta T ,$$

where ${m}_{w}$ and ${m}_{PCM}$ are the masses of water and PCM sample, ${m}_{t}$ is the mass of the test tube, and ${c}_{pw}$ and ${c}_{pt}$ are the specific heat capacities of water and the material of the test tubes. $\Delta T$ is the temperature step for partial enthalpy evolution. The meaning of coefficients $A$ and ${A}^{^{\prime}}$ is clear from Fig. 1.

### Experimental apparatus design and instrumentation

**Figure 2:**
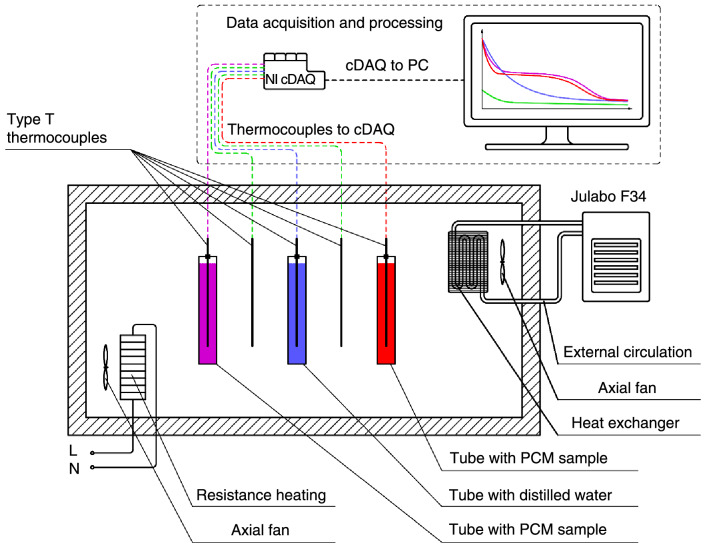
Schematic of the experimental apparatus for the T-history method including data acquisition and processing.

The PCM samples and the reference substance must be heated by the experimental apparatus to a temperature that is higher than the phase change temperature. The air must then be evacuated and cooled below the temperature of phase change. Figure 2 shows a schematic of the experimental setup. The heating was done with resistance heating elements controlled by a solid-state relay, and the regulation was done with an Arduino Uno and a PID regulation script. The air chamber was kept cool using a Julabo F34 ME cryostat. This device is connected to an external cooling circuit with a crossflow heat exchanger. To ensure consistent air circulation in the chamber, axial fans were positioned on the heat exchanger.

T-type thermocouples were used to measure the temperatures of the PCM samples, the reference material (distilled water), and the ambient air. In comparison with other types of thermocouples, T-type thermocouples were chosen for their great sensitivity and accuracy. However, they are constrained by the experiment's low maximum operating temperature (300–350 °C), which was not being attained. The thermocouples were connected to the NI thermocouple module, which was housed in the NI cDAQ-9174 chassis, which was directly connected to the computer. Data is collected using NI LabVIEW, and the experiment was subsequently evaluated using MathWorks MATLAB.

## Experimental heat storage

The experimental circuit was built and assembled with the goal of validating the results of the simulations as well as measuring methodologies and design solutions. The knowledge gained during the storage's design, assembly, and operation will be applied in future projects involving larger assemblies. The goal is to introduce a concept that would be competitive with the current, mostly water-based, sensible heat storages. Therefore, it has to be relatively simple from the technological point of view and made of affordable materials. It is also scalable in terms of overall heat storage capacity, temperature, and charge/discharge period.

Rubitherm's paraffin-based PCM RT35HC^29^ was used for storing. Its phase transition temperature is around 35 °C. The fundamental rationale for this decision was that working at lower temperatures is easier, and heat losses are smaller. Paraffins in general seem to be a good choice for PCM due to their long-term stability. Water as the heat transfer fluid (HTF) was a natural choice for the intended operation temperature.

### Storage design

**Figure 3:**
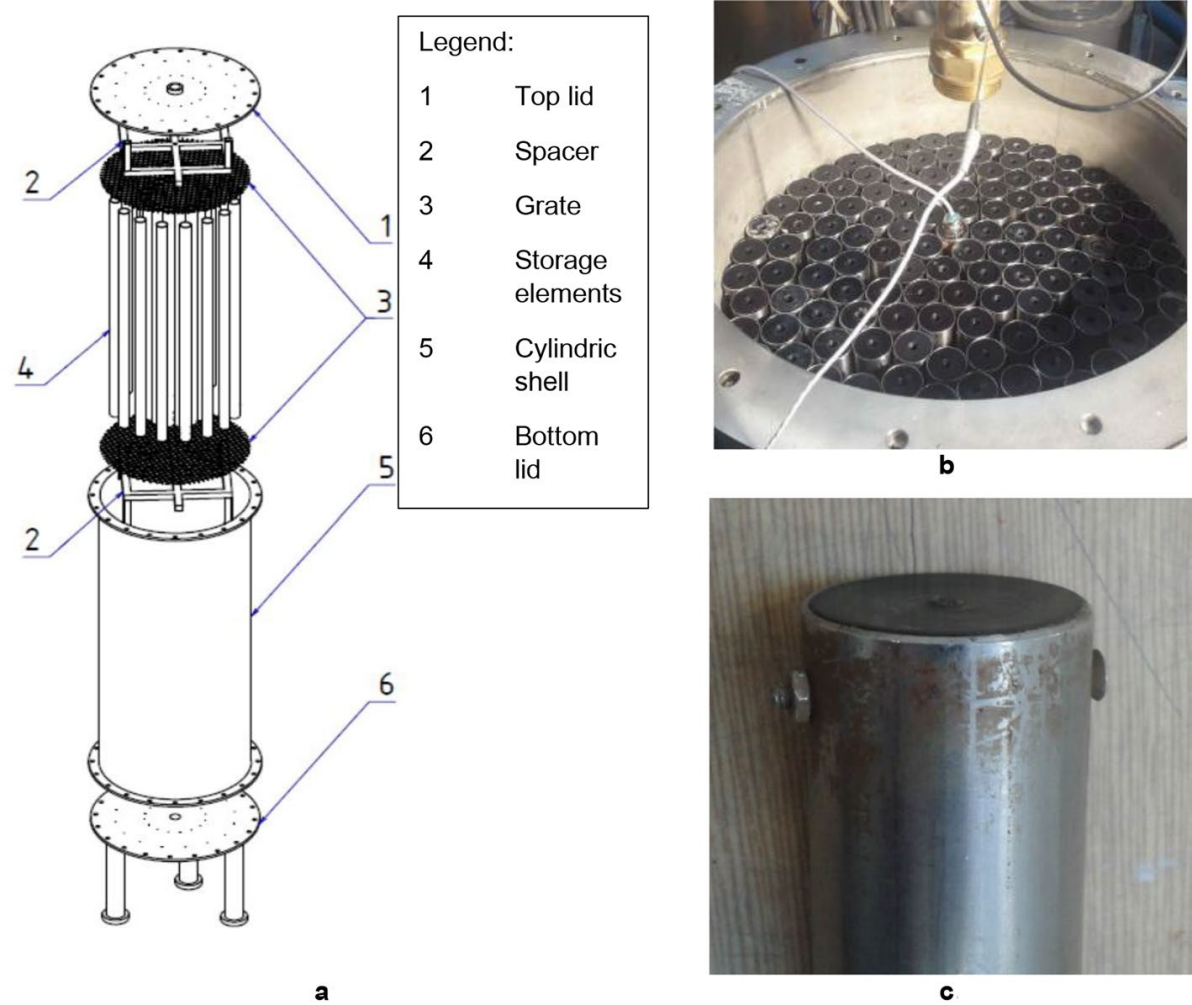
(**a**) The exploded diagram of an experimental PCM heat storage system. (**b**) The storage filled with cylindrical capsules. The capsule with PCM temperature measurement visible in the centre of the tank (**c**) The detail of a cylindrical capsule cap. Some of the caps did not remain tight after several cycles.

Figure 3a,b depict the experimental PCM heat storage. It was designed as a 125-L cylindrical vessel made of stainless steel. The container's inner diameter was 400 mm, and its height was 1000 mm. The lids were 8 mm thick, while the cylindrical wall of the container was 2 mm thick. Two necks on both the top and the bottom linked the storage to the system. To decrease heat loss, the tank was thermally insulated from the outside. The examined PCM was contained within stainless steel tubes with the outer diameter 30 mm, wall thickness 1 mm, and length 800 mm. Plastic caps with O-rings closed the tubes from both ends and a M3 bolt locked each cap in place (Fig. 3c). Small air pockets (about 20 mm in height) had been kept under the top caps during the filling to reduce pressure variations induced by PCM volume changes. The grates and spacers kept the storage elements in the desired position. In total, 143 containers had been placed into the storage, which was the maximum achievable number. About 80% of the tank cross-section was occupied by the PCM capsules.

The capsule cap design was easy to manufacture; however, it was not perfectly tight. After a few weeks of testing, a small amount of the PCM leaked to the water from about ten containers. The cap needs redesigning for a future storage because PCM leakage is unacceptable for a storage deployed to service in real conditions.

Table 1 summarizes the key characteristics of the storage. The PCM accumulates two thirds of the heat while its mass is only about one quarter of the whole. In contrast, cylindrical capsules contain only 5% of the energy while form over one third of the overall mass. Consequently, as little steel (or other construction materials) as possible should be used for a heat storage with high stored energy density. Especially cylindrical capsules seem to be needlessly robust saving material here would be effective.

**Table 1:** Parameters of the experimental heat storage. Stainless steel is highly ineffective for heat storage per unit mass.

	Mass (kg)	Stored energy (kWh) (between 25 °C and 55 °C)
PCM	45.24	4.008
Water	40.88	1.425
Cylindrical capsules (stainless steel)	80.55	0.303
Tank (including spacers and grates) (stainless steel)	55.56	0.208
Total	231.11	5.944

Comparison of latent heat storage designs is complicated since they are different in size, use different storage and construction materials, and were tested under different conditions. Some recently published designs and their main characteristics and testing conditions are listed in Table 2.

**Table 2:** Parameters and testing conditions of the previously published latent heat storage designs.

Authors	Design description	PCM (manufacturer)	T_min_/T_max_ (°C)	HTF flow rate (kg s^−1^ m^−3^)	Discharge time (minutes)	Energy density (kWh m^−3^)
This work	Cylindrically encapsulated PCM	RT35HC (Rubitherm)	25/55	1.00	125	47.6
Xu et al.^8^	Cylindrically encapsulated PCM	C58 (Climator)	35/65	0.35	360	43.7
Zauner et al.^18^	Shell-and-tube, PCM on shell side	HDPE (Ineos)	110/150	0.51	300	34.1
Zauner et al.^17^	Fin-tube, PCM on fin-side	HDPE (Ineos)	105/155	2.55	120	66.3

The temperature difference between the fully charged state and the fully discharged state and the PCM solidification temperature plays a crucial role in heat storage performance. A higher temperature difference allows using more sensible heat, and the higher the solidification temperature above the discharging temperature is, the more intensive is the discharging process. A higher flow rate of HTF decreases the time required for discharging as well. It is reasonable to norm the HTF rate by the size of a storage to get comparable results.

The storage analysed in this work had the PCM solidification temperature about 10 K above the inflowing HTF, and it was cooled down by 30 K over the discharging period. The storage presented by Xu et al.^8^ worked with the same temperature difference, but the PCM solidification temperature was about 20 K above the HTF inlet temperature. Despite this, the discharging process took almost three times more time. The reason was probably too large PCM capsules, and the lower flow rate played its role as well. The shell-and-tube design presented by Zauner et al.^18^ worked with higher temperature differences, and the PCM solidification temperature was about 15 K above the HTF inlet temperature. The discharging took long again, and the reasons are the same as in the previous case. The fin-tube design by Zauner et al.^17^ used the highest temperature range and the solidification-HTF inlet temperature difference of about 20 K. This significant temperature gradient, together with a rather complex design and high HTF flow rate, led to the shortest discharging time from all compared experiments. The LHTS presented in this article discharged very quickly, despite having the lowest solidification-inlet HTF temperature difference from all compared storages and relatively simple design. Fast charging/discharging is a desired feature of a LHTS since it increases its flexibility.

differenceA common motive for using PCMs is their superior heat energy density. Therefore, the overall heat energy density might be a good measure of the design effectiveness. The presented design has about 9% higher heat energy density then the design by Xu et al.^8^ despite they used PCM with higher latent heat per unit volume (298 MJ m^−3^ vs 211 MJ m^−3^). The design by Zauner et al.^18^ has even lower energy density, but it is fair to say that it is a hybrid latent-sensible storage and so energy density was not a priority. The highest energy density from the mentioned has the storage by Zauner et al.^17^. It is mostly because over 80% of the storage is occupied by PCM. Furthermore, a higher temperature difference plays some role. From the presented storages, it is the most complicated one from the manufacturing point of view and thus probably the most expensive. The storage presented in this work seems to have a remarkable energy density/design complexity ratio.

### Test circuit design and operation

**Figure 4:**
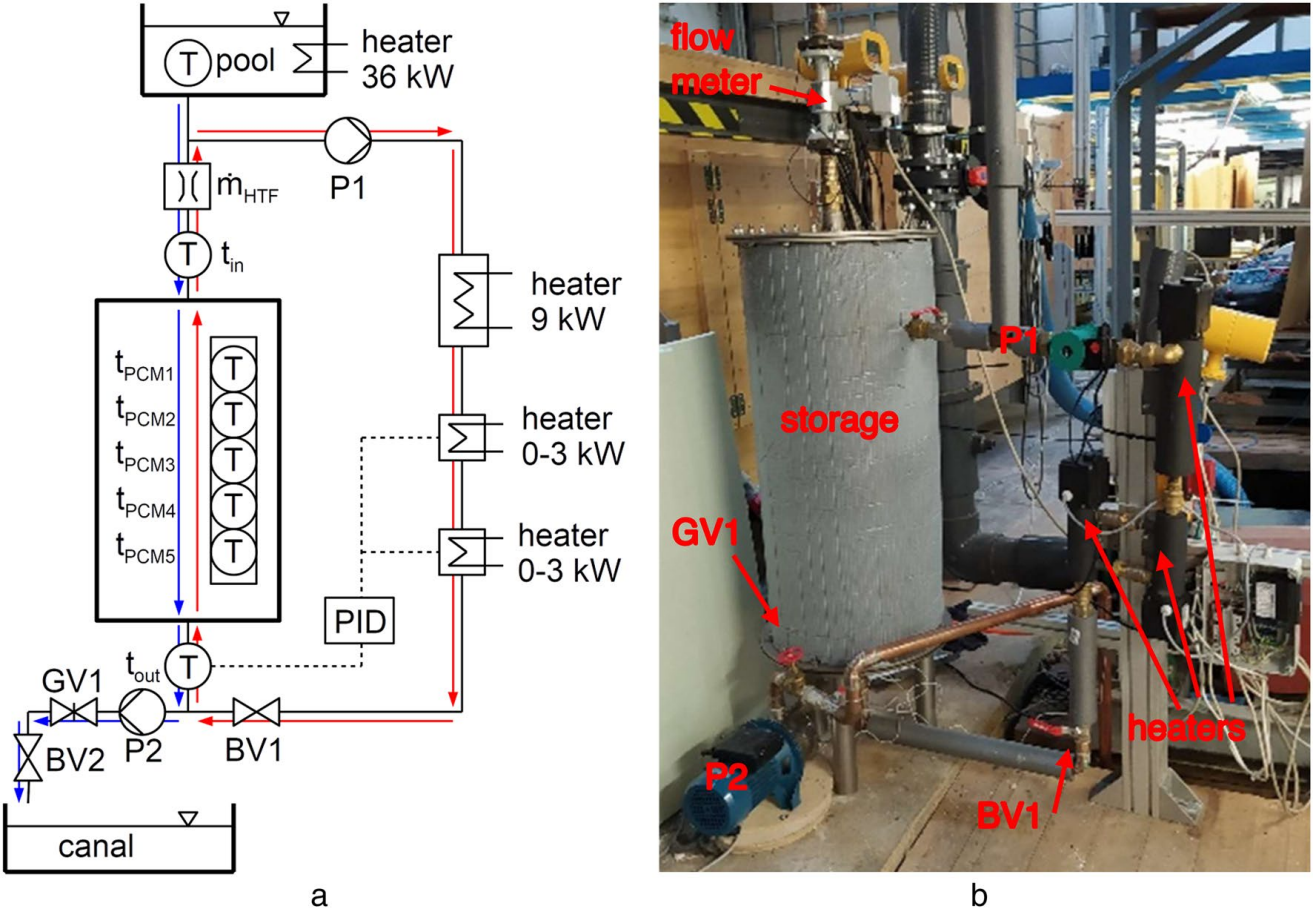
The diagram of the experimental circuit with the latent heat storage. (**a**) The HTF flow direction marked for charging (red arrows) and discharging (blue arrows) of the storage. (**b**) The real experimental circuit.

A circuit that allows both charging (melting PCM) and discharging (solidifying PCM) of the storage was built. Its schematic and physical realization depicts Fig. 4. In the current work, only discharging was investigated because it needs to be done quicker than charging, and consequently, it is the most critical process. Only the basic design and processes are described in this subsection. More details about the measurements and data acquisition contains the following subsection.

First, the storage had to be charged. Charging is indicated by the red arrows in the diagram in Fig. 4a. Water circulated in a closed loop driven by the pump P1. Three boilers heated the water, two with a power range of 0–3 kW (PID controlled) and one with a fixed power of 9 kW. The state of charge of the storage was checked by temperatures T1, T2, and T4 When the storage was charged, the boilers and the pump were switched off, and the ball valve BV1 was closed.

The discharging process indicates blue arrows in Fig. 4a. A large pool (25 m^3^) was employed as a water reservoir. The pool's water can be heated to a desired temperature of up to 60 °C utilizing heating elements with a power input of 36 kW. The pool was built in such a way that a consistent water level height is maintained throughout the trials, ensuring that there are no unwanted flow variations. The water flowed through the storage and its flow rate and temperatures at the inlet and on the outlet were recorded. The flow rate was adjusted by the pump (P2) in cooperation with the gate valve (GV1). The ball valve (BV2) allowed rapid changes in the flow rate needed for a well-defined start of the experiment. The canal located in the floor of the laboratory (45 m^3^) collected the water leaving the storage. At any point, the canal's water might be pumped back to the upper pool or to the sewer.

### Instrumentation and data acquisition

The experimental data collection and measuring system for heat storage was created for controlling the processes, monitoring the state of the storage, and collecting the data for analyses and model validation. The National Instruments system was used to collect and store the measured data.

The water flow measurement through the storage was needed for setting the required flow rate during the experiments and for storage’s power calculations. The installed magnetic induction flow meter ELIS IS1.110 was used to measure the water flow rate. The manufacturer declares its measured value uncertainty 0.5 %.

To achieve the desired temperature for the heat transfer fluid, the temperature of the pool water was measured. The temperatures at the storage's intake and exit were then measured, primarily for the purpose of calculating the storage's power. Pt100 sensors were used to record all these temperatures.

Another set of sensors was used to measure the PCM directly within a cylindrical capsule. To provide the most representative data, one was picked that was roughly in the middle of the tank. Five digital sensors were evenly spaced on a thread running across the cylinder's axis. The main reason for using digital sensors, despite their inferior precision in comparison to Pt100s (0.5 °C vs 0.1 °C), was their simple connection to the data acquisition system. Pt100s would have needed four wires each, while only three wires were necessary for all five digital sensors together. Too many wires could also influence heat transport within the capsule, thus making the measured data not representative for the other capsules.

The input of 0–3 kW boilers was controlled by an SSR relay with an input of 4–20 mA. The SSR relay was controlled by the installed PID controller based on the difference between the desired and actual temperature in front of the storage.

## Simulation

### PCM effective heat capacity model

When a typical PCM melts or solidifies, its enthalpy changes dramatically. Unlike pure crystalline solids, there is no abrupt shift at a specific temperature, but rather a progressive change over time. A mushy zone is a situation in which solid and molten material coexist^33^. Outside of this temperature range, the enthalpy is usually linearly proportional to temperature. Hysteresis within the mushy region is another essential property of almost all PCMs. PCMs melt at a slightly greater temperature than they solidify at (approximately 1–2 K for paraffin-based PCMs).

The liquid phase mass fraction defined as

2 $$\xi =\frac{{m}_{l}}{{m}_{l}+{m}_{s}},$$

is used to describe the condition of a PCM. In the latter expression, ${m}_{l}$ is the mass of the liquid phase and ${m}_{s}$ is the mass of the solid phase. In the mushy, zone it is a continuous function of temperature, and it reaches zero for fully solidified PCM and unity for fully melted PCM.

The specific enthalpy as a function of temperature can be written as

3 $$h\left(T\right)={\int }_{{T}_{0}}^{T}\xi \left(\tau \right){c}_{l}+\left[1-\xi \left(\theta \right)\right]{c}_{s}\text{d}\theta +\left[\xi \left(\theta \right)-\xi \left({T}_{0}\right)\right]{\Delta h}_{F} ,$$

where ${c}_{l}$ and ${c}_{s}$ are specific heat capacities of the liquid and solid phases (assumed to be constant), respectively, ${\Delta h}_{F}$ is the specific enthalpy of fusion (latent heat), and ${T}_{0}$ is the temperature for which the enthalpy is defined as zero. The effective specific heat capacity of a PCM is then

4 $${c}_{eff}\left(T\right)=\xi \left(T\right){c}_{l}+\left[1-\xi \left(T\right)\right]{c}_{s}+\frac{\text{d}\xi \left(T\right)}{\text{d}T}{\Delta h}_{F} .$$

The selection of the liquid phase mass fraction function is a crucial step. Probability distribution functions are a nice class of functions to choose from in general. They grow indefinitely from zero to unity, and their shape is controlled by a small number of parameters^34^. Nonlinear interpolation techniques are commonly used to find the parameters. The enthalpy-temperature relationship is not always straightforward. In such instances, a linear combination of additional distribution functions, which increases the number of modifiable parameters, may be useful.

For the material RT35HC, used in the experiments, a linear combination of the Gumbel minimum distribution functions was employed:

5 $$\xi \left(T\right)={w}_{1}{\xi }_{1}\left(T\right)+\left(1-{w}_{1}\right){\xi }_{2}\left(T\right),$$

6 $${\xi }_{i}\left(T\right)=1-\text{exp}\left[-\text{exp}\left(\frac{T-{\mu }_{i}}{{\beta }_{i}}\right)\right] ; i=\text{1,2} .$$

This study only discusses the solidification process. The only difference would be that extra parameters would have to be fitted if melting was also handled. The parameters were fitted using partial enthalpy data from the T-history experiment. It is important to understand that partial enthalpy does not equate to effective heat capacity. The relationship between these two numbers is as follows:

7 $${h}_{part}\left(T\right)={\int }_{T-\frac{\Delta T}{2}}^{T+\frac{\Delta T}{2}}{c}_{eff}\left(\theta \right)\text{d}\theta ,$$

where $\Delta T$ is the temperature step between two neighbouring partial enthalpy values. Since $\Delta T$ is $1 \text{ K}$ in our case, the numerical values of the partial enthalpy are virtually the same as the specific effective heat capacity values for the same temperature. However, for a different temperature step, the numerical results would be different. The least squares method was used to find the parameters by comparing the measured and modelled partial enthalpy.

### Storage model

A storage model was created as a tool for analysing storage performance. The model's simplicity and computing speed were prioritized during development. The goal was to develop a model that could be used not just on its own, but also as part of a larger heat distribution system that included various heat suppliers and consumers, as well as multiple instances of heat storage.

**Figure 5:**
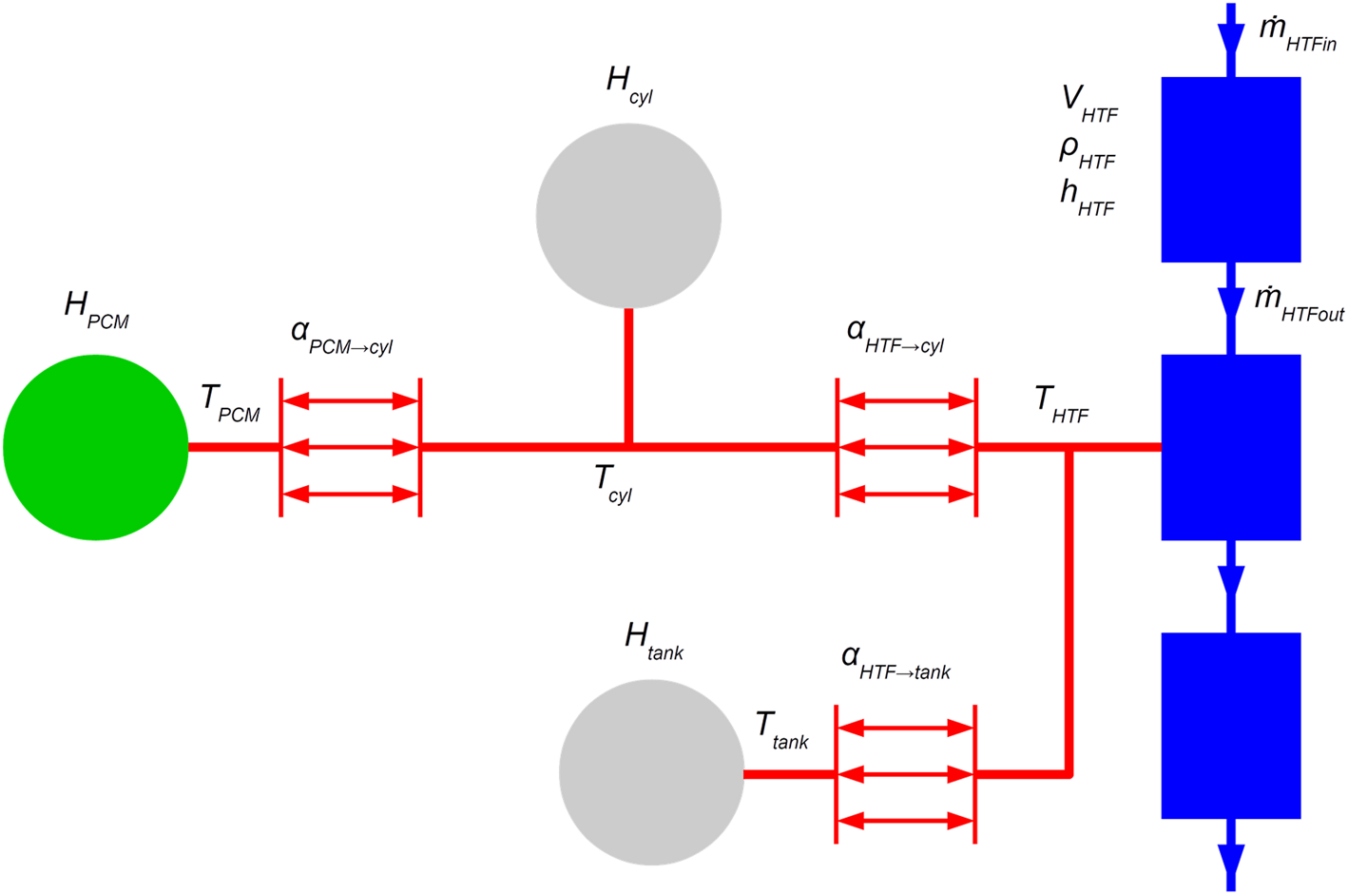
Topology of the lumped latent heat storage model with the HTF (blue rectangles), capacity of steel (grey circles), capacity of PCM (green circle), and heat transfer (red arrows) elements.

Due to its computing efficiency, a lumped parameter technique was chosen because it permits simulation of processes that take hours in the real world within seconds. Figure 5 displays the model's topology. Heat transfer fluid volumes, heat capacitors, and heat transfer components are the three categories of elements in the model.

In the model, three heat transfer fluid volumes are connected in series. The top and bottom volumes represent the volumes above and below the heat transmission zone, respectively. These volumes have no heat transfer represented in them, and the HTF flows into and out of the model through them. The intermediate volume, which symbolizes the water between the cylinders filled with the PCM, is where all heat transfer occurs. Due to a relatively narrow temperature range, the thermophysical characteristics of the HTF (water) are assumed to be constant. The volume of HTF is calculated using the known dimensions.

Rate of change of HTF mass in a volume can be described by the following equation

8 $${V}_{HTF}\frac{\text{d}{\rho }_{HTF}}{\text{d}t}={\dot{m}}_{HTFin}-{\dot{m}}_{HTFout} ,$$

where ${\rho }_{HTF}$ is the density of HTF in volume ${V}_{HTF}$ and ${\dot{m}}_{HTFin}$ and ${\dot{m}}_{HTFout}$ are the mass flow rates into the volume and out of it, respectively. This equation considers possible changes of mass within a volume due to HTF density change; the volume is considered to remain constant.

Total enthalpy in a HTF volume is described by

9 $${V}_{HTF}\frac{\text{d}({\rho }_{HTF}{h}_{HTF})}{\text{d}t}={\dot{m}}_{HTFin}{h}_{HTFin}-{\dot{m}}_{HTFout}{h}_{HTF}+{\dot{Q}}_{in} ,$$

where ${h}_{HTF}$ is HTF specific enthalpy and the terms on the right-hand side of the equation give the convective transport of enthalpy and heat transfer across the volume’s boundary. The system is closed by relations in form ${\rho }_{HTF}={\rho }_{HTF}(T)$ and ${h}_{HTF}={h}_{HTF}\left(T\right).$

There are also three heat capacitors which represent the steel tank, steel cylinders containing the PCM, and the PCM itself.

The general equation for the rate of change in enthalpy of a heat capacitor $H$ is

10 $$\frac{\text{d}H}{\text{d}t}=C\frac{\text{dT}}{\text{d}t}={\dot{Q}}_{in} ,$$

where $C$ is the heat capacity of the component. The heat capacity of the steel components is defined by their mass and specific heat capacity of stainless steel (constant due to a narrow temperature range). Heat capacity of the PCM is temperature dependent as described in section 4.1. It is assumed here that the process is isobaric and so the transferred heat rate into the component is equal to the rate of change in enthalpy.

Heat flux between the components is governed by three heat transfer components that interconnect the heat capacities and the middle HTF volume. In general, Newton’s cooling law describes the heat flux ${\dot{Q}}_{a\to b}$ between components $a$ and $b$ in the form

11 $${\dot{Q}}_{a\to b}={\upalpha}_{a\to b}{\text{A}}_{ab}\left({T}_{a}-{T}_{b}\right),$$

where ${\upalpha}_{a\to b}$ is the respective heat transfer coefficient, ${\text{A}}_{ab}$ is the contact area between the two components, and ${T}_{a}$ and ${T}_{b}$ are temperatures of the two components. The area can be calculated using the geometry, and the temperature can be calculated using the states of the interconnected components. A correct value for the heat transfer coefficient has yet to be assigned.

The most important part is the interaction between the HTF and the PCM. First, there is a heat transfer between the surface of the steel cylindrical containers and the HTF which flows along them. Heat transfer coefficient is in this case depends on Nusselt number $\text{Nu}$, the heat conductivity of HTF ${\lambda }_{HTF}$, and the diameter of the cylindrical containers $d$ in the form

12 $${\upalpha}_{HTF\to cyl}=\frac{2\cdot \text{Nu}\cdot {\lambda }_{HTF}}{d} .$$

The correlation used for finding $\text{Nu}$ for laminar flow in a channel bounded by three curved walls is^35^

13 $$\text{Nu}=3.66+4.12{\left(\frac{2{D}_{h}}{d}-0.205\right)}^{0.569} ,$$

where ${D}_{h}$ is the hydraulic diameter defined as

14 $${D}_{h}=\frac{4\left[{a}^{2}\frac{\sqrt{3}}{4}-\frac{\pi {d}^{2}}{8}\right]}{\frac{\pi d}{2}} ,$$

where $a$ is the distance between axes of the cylinders (the cylinders are assumed to be in a regular triangular pattern). The correlation in Eq. (13) is applicable for laminar flow between the cylinders, which is valid for all presented cases.

While the heat transfer coefficient on the outside of the cylinders is relatively simple to calculate using a Nusselt number correlation, the situation on the inner surface of the cylinder is more difficult to calculate because the process is complex and the quality of the process changes over time.

Because all PCM is liquid at the start of the discharge operation, free convection in a cylindrical capsule is the primary heat transmission mechanism. As the heat from the cylinder is gradually removed, a layer of solidified PCM forms on the cylinder's inner wall. Because this layer contains just heat conduction, the wall's heat resistance increases. The thickening layer also reduces the amount of space available for liquid phase flow. Consequently, the heat transmission intensity decreases. When there is no longer any liquid phase, heat conduction is the sole option.

From the description of the process, it shows that the quality of the heat transfer is greatly affected by the mass fraction of the liquid phase $\zeta$. It will be quite intensive for high liquid content fraction and not so intensive for the opposite case. A simple way to capture this behaviour is using the linear relation

15 $${\alpha}_{PCM\to cyl}=\left(1-\upxi \right)\cdot {\upalpha}_{solid}+\xi \cdot {\upalpha}_{liquid} ,$$

where ${\upalpha}_{solid}$ is the heat transfer coefficient for the case of fully solidified PCM and ${\upalpha}_{liquid}$ the heat transfer coefficient for the case of fully melted PCM. The question remains, how to determine the appropriate values for these parameters.

The optimum method would be to develop a Nusselt number correlation for free convection within a cylindrical cavity. Unfortunately, such a correlation seems not to be available. Therefore, it was decided to begin the simulations with a rough estimate of the heat transfer coefficients and fine-tune them until the experimental data are fit.

For the initial guess of the heat transfer coefficient, we assumed the Nusselt number for fully melted within the cylinder 5 and for fully solidified 0.5. The characteristic dimension is the inner diameter of the cylindrical tube and the heat conductivity of the PCM was given by the manufacturer. The heat transfer coefficient estimates obtained from these values are close to the values from the tuning with the experimental data.

The heat transfer coefficient between the heat transfer fluid and the steel tank is the last heat transfer coefficient to be computed for the model. In comparison to the capacity of the PCM or the water content of the tank, the steel tank's heat capacity is comparatively low. As a result, this parameter has no bearing on the model's outcome. The middle section of the tank has forced convection where water flows along the cylindrical capsules, while the mixing volumes above and below the cylinders have a blend of free and forced convection. The flow velocities near the tank walls are predicted to be extremely low, resulting in a low heat transfer coefficient. Different values from $50 \text{ W }{\text{m}}^{-2} {\text{K}}^{-1}$ to $300 \text{ W }{\text{m}}^{-2} {\text{K}}^{-1}$ were tested with hardly noticeable differences in the simulations. The presented results are for ${\upalpha}_{HTF\to tank}=100 \text{ W }{\text{m}}^{-2} {\text{K}}^{-1}$.

## State of charge

In this work, two SoC evaluation methods are used, both based on the temperature measurement. For both methods, a reference heat content value has to be defined. Here it is defined as the difference in the enthalpy of all components of the storage between the minimal and the maximal temperature

16 $$\Delta {H}_{max}={\int }_{{T}_{min}}^{{T}_{max}}\left({c}_{eff}\left(\theta \right)\cdot {m}_{PCM}+{c}_{HTF}\cdot {m}_{HTF}+{C}_{cyl}+{C}_{tank}\right)\text{d}\theta .$$

If the necessary parameters in eq. 16 were not known, an alternative for obtaining $\Delta {H}_{max}$ would be charging or discharging the storage between temperatures ${T}_{min}$ and ${T}_{max}$ and integration of the power output.

The first method we use for getting SoC is based on the integration of storage power in time. The formula for the process of discharging starting from a fully charged storage is

17 $${SoC}_{P}(\text{t})=1-\frac{{\int }_{0}^{t}{\dot{m}}_{HTF}\cdot \left({h}_{HTFout}-{h}_{HTFin}\right)d\tau }{\Delta {H}_{max}}.$$

The advantage of this method is that it simply requires temperature readings at the inlet and outflow, as well as the HTF flow rate. It is also not necessary to understand the PCM's temperature-enthalpy dependence. On the other hand, measurement mistakes and heat losses accumulate over time, causing a drift from the correct value. As a result, it does not appear to be suited for longer periods of time, and it should be reinitialized once the storage state is known (either fully charged or fully discharged).

The second method is based on calculating the enthalpy of all essential storage components. It is for the explored storage

18 $${SoC}_{H}=\frac{\sum_{i}{\int }_{{T}_{min}}^{{T}_{PCM}(i)}{c}_{eff}(i)\cdot {m}_{PCM}(i)\text{d}\theta +{\int }_{{T}_{min}}^{{T}_{HTF}}{c}_{HTF}\cdot {m}_{HTF}\text{d}\theta +{\int }_{{T}_{min}}^{{T}_{cyl}}{C}_{cyl}\text{d}\theta +{\int }_{{T}_{min}}^{{T}_{tank}}{C}_{tank}\text{d}\theta }{\Delta {H}_{max}}.$$

The benefit of this method is that it is only dependent on the status of the storage and, as a result, the energy losses in the system are automatically considered. On the other hand, we need to know the temperatures at numerous sites throughout the storage, particularly within the PCM with large temperature gradients, as well as the enthalpy dependence on the temperature of all components, including the PCM. Five temperature zones representing equal sections of the PCM are evaluated for evaluation based on experimental data. Temperatures ${T}_{HTF}$, ${T}_{cyl}$, and ${T}_{tank}$ are not measured directly. The average temperature of the outlet and the inlet of the storage was used in their place successfully.

## Results and discussion

This section presents the results of the experiments (both T-history and storage discharge), validation of the numerical model, and evaluation of SoC. Two regimes with different HTF flow rate were investigated (roughly 0.12 kg s^–1^ and 0.5 kg s^–1^). At the start, the storage was at the uniform temperature of 55 °C and it was discharged by water at 25 °C in both cases.

### Specific partial enthalpy and effective heat capacity model

Rubitherm proclaims 240 kJ kg^−1^ between 27 °C and 42 °C for RT35HC, with a 7.5 percent uncertainty^29^. The provided PCM's certificate of analysis stated that it had a specific heat storage capacity of 258 kJ kg^−1^ at a temperature differential of 15 °C. There is also a more thorough partial enthalpy distribution for 1 °C temperature steps, which peaks about 35 °C. Three layer calorimeter measurements yielded these results.

**Figure 6:**
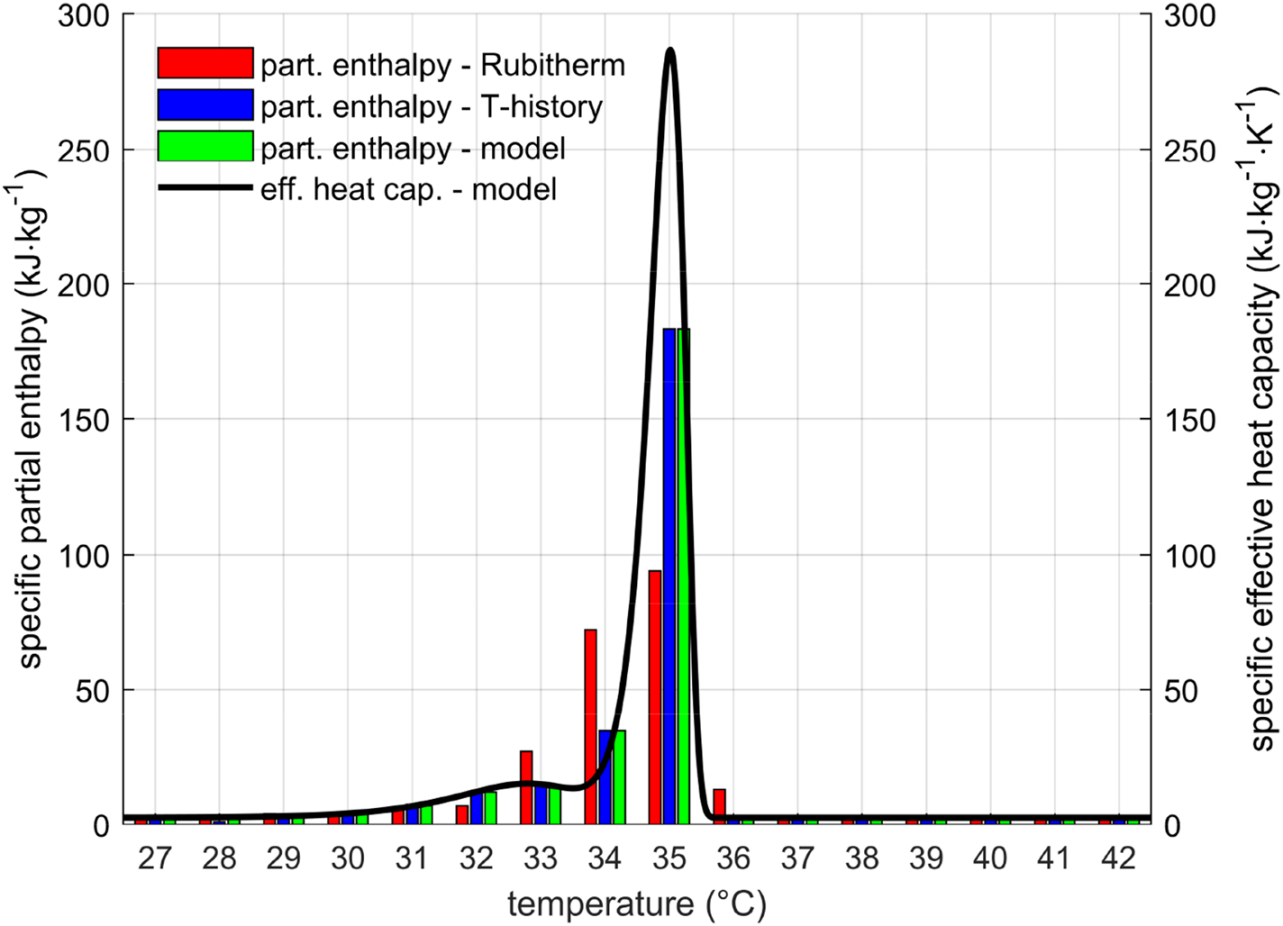
Partial enthalpy and specific effective heat capacity for solidification of RT35HC. Partial enthalpy and heat capacity are not the same, although the numerical values are often very close.

The effective heat capacity model according to Eq. (4) was successfully fitted to the measured data. Figure 6 illustrates the values of specific partial enthalpy of RT35HC together with its effective heat capacity. The data obtained by T-history method show a sharp peak in partial enthalpy, while the data provided by Rubitherm have partial enthalpy distributed more evenly. In the compared range from 27 °C to 42 °C was the measured specific enthalpy difference 283 kJ kg^−1^, which was about 10 percent more than declared by Rubitherm. The reason for this difference is unknown.

### Measured water and PCM temperatures

**Figure 7:**
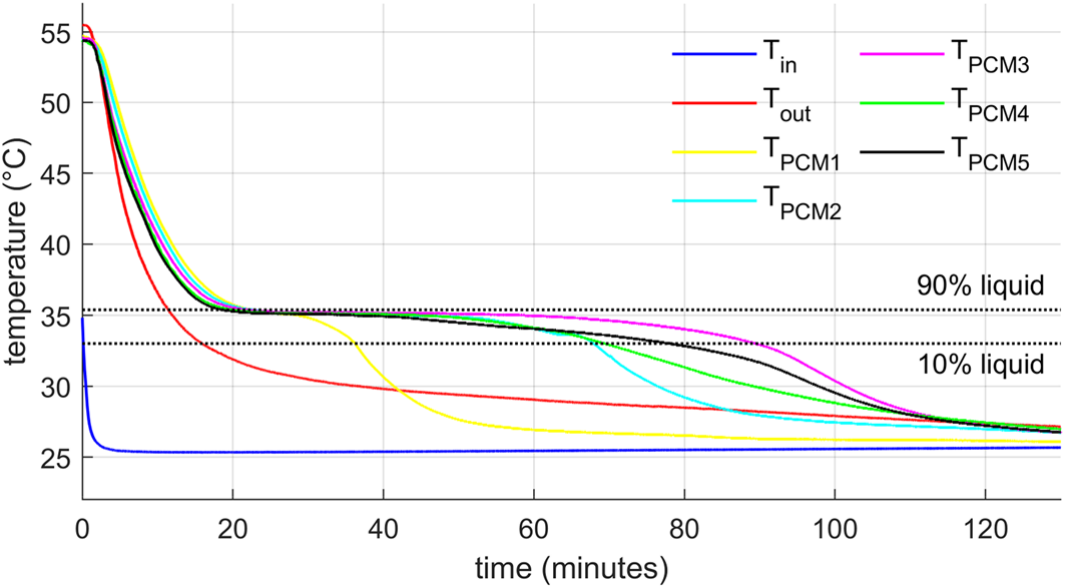
HTF (inlet and outlet) and PCM (five positions in the capsule from top to bottom) temperature evolution for lower HTF mass flow rate. The PCM phase transition zone marked by 10% and 90% liquid ratio temperatures.

The graph in Fig. 7 displays the evolution of recorded temperatures of the HTF and PCM during the experiment with the lower flow rate. The higher flow rate yielded very similar results, thus not presented here. The inflow temperature stayed constant throughout the experiment. The higher temperature during the first few minutes of discharge was caused by heated pipe sections and warm water residues in the system upstream the storage.

Five temperatures were measured in the axis of one of the cylindrical containers with the PCM. The sensors were equally spaced with ${T}_{PCM1}$ beingthe top one and ${T}_{PCM5}$ being the bottom one. Initially, all PCM temperatures followed the outlet temperature and dropped rapidly to the phase change temperature (phase change region highlighted by 10% and 90% of the liquid phase in the graphs). The temperature descended from top to bottom despite that the cold water was distributed from the top. This temperature stratification indicates a relatively strong natural convection in the capsule during this phase. Once the temperature reached the phase change zone, it remained almost constant for some time, while the outlet water temperature was gradually descending. It is worth noting that there was no visible subcooling, the temperature decreased monotonously. Solidification of the PCM stopped the natural convection and consequently, the first part which fully solidified was the top one, where the heat transfer intensity was the greatest. The remaining sections followed as well, while the central one solidified as the last. After solidification in each measured location, the temperature decreases relatively steeply, but not like in the beginning. This is not only due to the smaller temperature difference but primarily because conduction in solids is a far less effective heat transport mechanism than convection in liquids.

### Model validation

The heat storage model presented in Sect. 4 was validated against the measured data. Parameters of the model (masses, volumes) were taken from the geometry of the experimental storage. Inputs to the model were the measured flow rate and the water temperature at the inlet. The comparison between the modelled outlet temperature and the measured outlet temperature indicated the level of agreement of the simulation.

**Figure 8:**
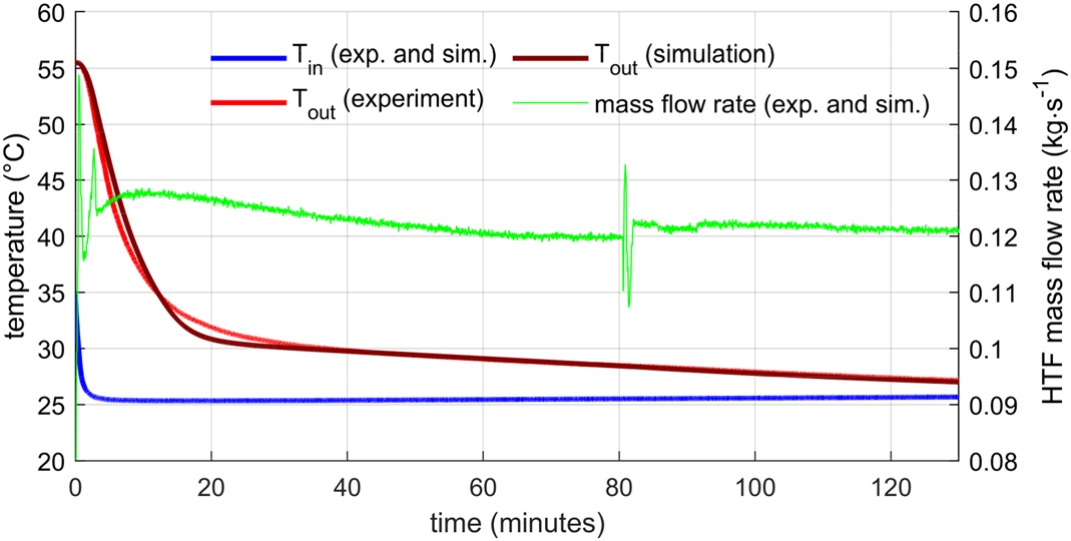
HTF temperature evolution—comparison of the numerical model with the experimental data for lower HTF flow rate. The greatest discrepancy was between 15 and 25 min.

**Figure 9:**
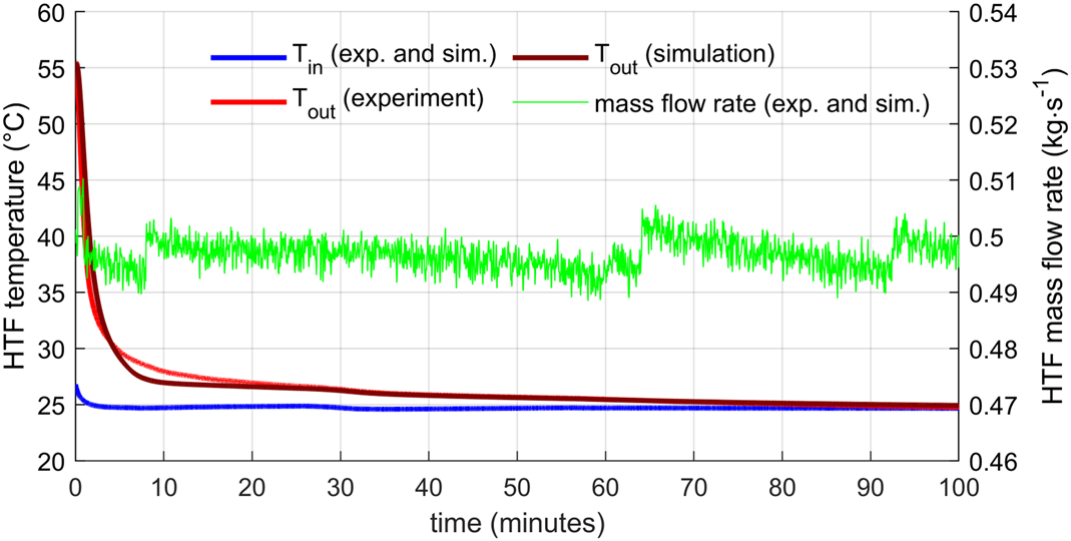
HTF temperature evolution—comparison of the numerical model with the experimental data for higher HTF flow rate. The greatest discrepancy was between 5 and 15 min.

The model’s results present Fig. 8 for the lower flow rate and Fig. 9 for the higher flow rate. There are two phases: the initial characterized by a steep drop in the outlet temperature and the later with a gradual outlet temperature descend. The overall agreement is very good, slightly better for the lower flow rate. The greatest difference in the outlet temperature is in the transition stage between the initial fast drop and the shallow descend later. Exact causes of the discrepancies are unknown to the authors. One might be that the model is too crude, not capturing all phenomena occurring in the storage properly. However, the disagreement is not critical for the intended purpose of the model. Size of the virtual storage can vary and the used PCM can change to reach different temperature levels if it has similar properties (density, heat conductivity), which is the case for most paraffin-based PCMs.

The mass flow rate was controlled manually by the gate valve (GV1). For the lower flow rate (Fig. 8), the regulation was not perfect. In the beginning and then at the time around 82 min, flow oscillations occurred. The origin of disturbances that caused these oscillations is unknown. Some hydraulic parameters of the system had to shift suddenly. However, even the most significant oscillations do not change the flow rate more than 15 % of the average flow rate, and there is no visible effect on the outlet temperature. Moreover, the measured mass flow rate is taken directly as one of the inputs to the numerical model, so the flow fluctuations do not affect the comparison between the experiment and the model. For the higher flow rate (Fig. 9), the flow rate remains within 2 % of the average flow rate without any distinct oscillations.

### Power and state of charge

**Figure 10:**
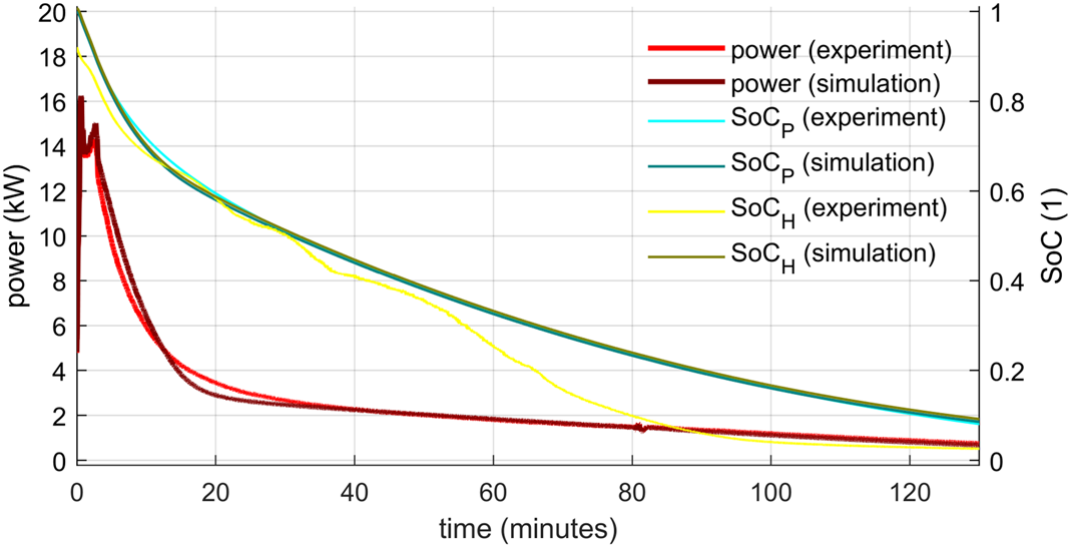
Power and SoC evolution—comparison of the numerical model with the experimental data for lower HTF flow rate.

**Figure 11:**
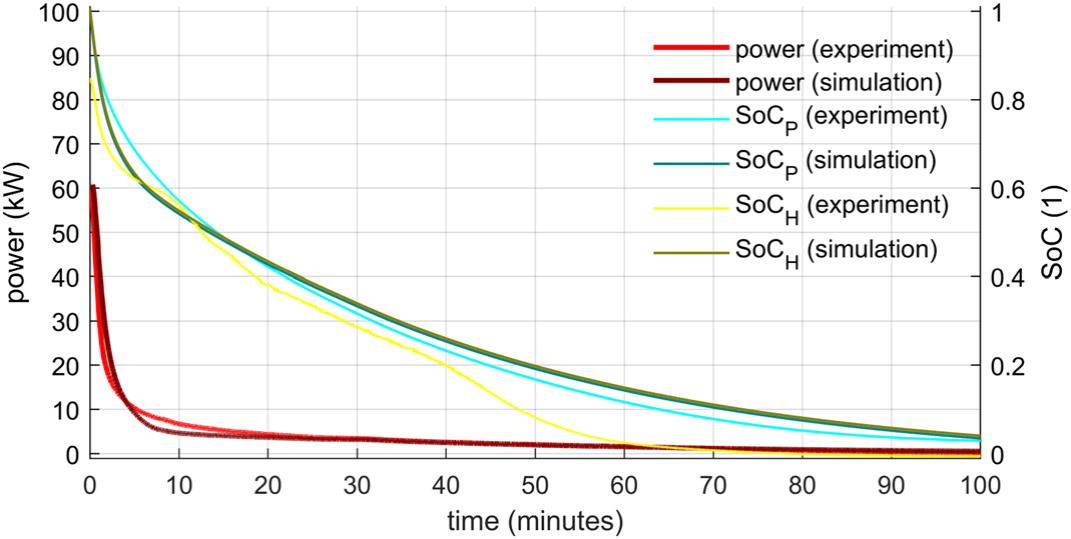
Power and SoC evolution—comparison of the numerical model with the experimental data for higher HTF flow rate.

Complex information about the storage performance provides the heat power output of the storage given by

19 $$\dot{Q}={\dot{m}}_{HTF}\cdot \left({h}_{HTFout}-{h}_{HTFin}\right)$$

and SoC defined by Eqs. (16 and 17). These quantities depict the graphs in Figs. 10 and 11.

The authors identified two stages in the power output of both analysed cases: The steep drop from a relatively high initial value and the gradual descend. The initial power peak was directly proportional to the flow rate. because the inflowing cold water replaced the warm water within the storage. The water contained in the storage accumulated about 25% of the storage’s total energy for the chosen temperature range, and most of it was released during the first phase. The second phase characterized a gradual power descend as the heat from the PCM was released slower and slower due to PCM solidification. This phase takes much longer compared to the first one and thus it is critical for the storage operation.

For both stages, higher power was reached for the higher flow rate. However, a rapid energy release cannot last very long. The lower flow rate power output surpassed the higher one in just four minutes and stayed on top for ten minutes. The situation repeated itself after 47 min from the start of the experiment. Since heat storages are supposed to operate continuously for at least a couple of hours with stable output, it seems that lower flow rates are more suitable. Another argument is that lower flow rates result in higher temperature differences that are more applicable in heating systems. A gradual flow rate growth could make the power output more uniform, but an additional source of heat (another storage or heater) would have to reheat the outflowing HTF in later periods.

The SoC was evaluated by both presented approaches (Eqs. 16 and 17) using the data from the experiments and from the simulations. The application to the simulation data yielded virtually the same results for both methods. The power integration method (SoC_P_) used on the experimental data led to the result very close to these from the simulation (especially in the lower flow rate). However, the enthalpy method (SoC_H_) calculations from the experimental data differ from the rest. First, the fully charged storage does not start at unity. The reason is that the inner water temperature is taken as the average of the inlet and outlet. It does not represent the reality correctly, especially in the beginning. The authors do not consider this a major problem since the discrepancy disappears in several minutes. As a greater problem has to be addressed the significant decrease of SoC that happened about halfway through the experiments. This decrease is associated with the solidification of the PCM that is indicated by temperature measurements inside the capsule. The authors have two possible explanations of the discrepancy. It might happen because the sensors in the capsule did not represent the true state of the PCM. The temperature-enthalpy dependency might not be precise, or the sensors were not in the axis of the cylinder. The other possibility is that the chosen measured capsule did not represent an “average” capsule. Possibly, the water flow around the capsules was more intense in the center than near the wall of the storage. The measured capsule would be then cooled down faster, and that is what the data indicate. A combination of both factors is not ruled out as well.

The duration of discharging was evaluated based on SoC_P_ from the experiments. The results are presented in Table 3. A significant shortening of the time required for discharge was accomplished by increased flow rate. Four-time greater flow rate led to cutting the time to about one-third for lower degrees of discharge (higher SoC) and to about one half for deeper discharges. However, as already stated, higher flow rates generate lower inlet/outlet difference in temperature with low utility and a reheat is necessary.

**Table 3:** Times from the start of discharging and inlet/outlet temperature differences for chosen SoC values.

SoC_P_ (experiments)	Lower flow rate (0.12 kg s^−1^)		Higher flow rate (0.5 kg s^−1^)	
	Time (min)	T_out_ − T_in_ (°C)	Time (min)	T_out_ − T_in_ (°C)
0.8	5.9	16.7	2.0	8.8
0.5	31.4	5.0	14.3	2.6
0.1	121.5	1.8	64.0	0.7

## Summary

The presented work deals with a complex task of design, testing, numerical modelling, and monitoring of a latent heat storage that can work with heat sources with unstable or irregular heat supply, such as solar collectors or combined-heat-power units.

The aim of the storage design was stored energy density per unit volume, fast charging/discharging, manufacturing simplicity, and low production cost. The laboratory scale heat storage was assembled and tested. It performed well although some deficiencies were found, especially the cylinder capsule sealing must be reworked. However, the potential of the general idea of cylindrical capsules containing PCM is promising and yields good parameters in comparison to previously published designs.

PCM state and specific heat capacity were modelled as temperature dependent with Gumbel distribution function. The model parameters were fitted to the data of specific partial enthalpy provided by Rubitherm. The specific heat capacity became a crucial part of the latent storage model. This lumped parameter model considered all parts of the storage relevant for heat accumulation and the HTF flow through the storage. The complex heat transfer during PCM solidification was solved with a heat transfer coefficient dependent on the state of the PCM. The model was successfully tuned and validated with the experimental data. The model is computationally inexpensive and can be incorporated into models of larger systems.

The experimental investigation included flow rate measurements of the HTF and temperature measurements of the HTF and PCM during storage discharge. The authors identified two stages: a very rapid initial drop of outlet temperature and power associated with the water replacement, and a gradual descend of outlet temperature and power caused by slow energy release from the PCM. PCM temperature data confirmed the complexity of PCM solidification within the capsules with natural convection in liquid and heat conduction in solid. The collected data also allowed the evaluation of the dependency of discharge time on the flow rate. Higher HTF flow rate shortened the discharge time significantly in exchange for low temperature differences between the inlet and the outlet.

Two ways of evaluation of the state of charge (SoC) were used with both experimental and modelled data. The only one that differentiated from the rest was the SoC evaluated from the overall storage enthalpy from the experimental data. A possible reason is that the measurements did not capture the storage state properly. The sensor placement should probably be different.

Even though the experiments were conducted on a laboratory scale, the authors are confident that the results can be applied to larger projects. However, other aspects that were not mentioned in this work must be resolved first for a technically and economically successful service of a heat storage. One of them is the choice of a suitable PCM, especially regarding phase change temperature and long-term stability. Another one is a power control strategy that allows effective heat storage when there is surplus and its back release in times of shortage.
